# S100. EFFECTS OF CANNABIS USE ON BODY MASS, FASTING GLUCOSE AND LIPIDS DURING THE FIRST 12 MONTHS OF TREATMENT IN SCHIZOPHRENIA SPECTRUM DISORDERS

**DOI:** 10.1093/schbul/sby018.887

**Published:** 2018-04-01

**Authors:** Frederika Scheffler, Sanja Kilian, Bonga Chiliza, Laila Asmal, Lebogang Phahladira, Stefan du Plessis, Martin Kidd, Robin Murray, Marta Di Forti, Soraya Seedat, Robin Emsley

**Affiliations:** 1 Stellenbosch University; 2 Institute of Psychiatry, King’s College, London

## Abstract

**Background:**

Acute cannabis use stimulates appetite, while general population studies suggest that chronic use is associated with reduced risk of obesity and other cardiometabolic risk factors.

**Methods:**

In this study, we investigated changes in body mass index (BMI), fasting blood glucose and lipids, and rates of metabolic syndrome risk factors in cannabis users vs. non-users in 109 minimally treated patients with first-episode schizophrenia, schizophreniform or schizo-affective disorder who were treated according to a standardized treatment regime with depot antipsychotic medication over 12 months. Participants underwent repeated urine toxicology tests for cannabis and those testing positive at any time during the study (n=40), were compared with those who tested negative at all time points (n=69).

**Results:**

There was a significant group*time interaction effect (p=0.002) with the cannabis negative group showing a greater increase in BMI than the cannabis positive group, after adjusting for age, sex, methamphetamine use and modal dose of antipsychotic. There were no group*time interaction effects for fasting blood glucose or lipids. Post hoc tests indicated significant increases in fasting blood glucose and triglycerides and a decrease in high-density lipoprotein cholesterol for the cannabis negative group, with no significant changes in the cannabis positive group. Rates of metabolic syndrome did not differ significantly between groups. However, more cannabis negative patients had elevated waist-circumference at endpoint (p=0.003).

**Discussion:**

Although other indirect effects such as dietary neglect and smoking may be contributory and could explain our findings, it may be that chronic cannabis use directly suppresses appetite, thereby preventing weight gain in users.

